# Clinically diagnosed childhood asthma and follow-up of symptoms in a Swedish case control study

**DOI:** 10.1186/1471-2296-6-16

**Published:** 2005-04-21

**Authors:** Eduardo Roel, Åshild Faresjö, Olle Zetterström, Erik Trell, Tomas Faresjö

**Affiliations:** 1 Department of Health and Society/General Practice and Primary Care, Faculty of Health Sciences, University of Linköping, SE-581 83 Linköping, Sweden; 2 Department of Molecular and Clinical Medicine /Allergy Centre, Faculty of Health Sciences, University of Linköping, SE-581 83 Linköping, Sweden

## Abstract

**Background:**

Childhood asthma has risen dramatically not only in the western societies and now forms a major and still increasing public health problem. The aims of this study were to follow up at the age of ten the patterns of asthma symptoms and associations among children with a clinically diagnosed asthma in a sizeable urban-rural community and to in compare them with demographic controls using a standardised questionnaire.

**Methods:**

In a defined region in Sweden with a population of about 150 000 inhabitants, all children (n = 2 104) born in 1990 were recorded. At the age of seven all primary care and hospital records of the 1 752 children still living in the community were examined, and a group of children (n = 191) was defined with a well-documented and medically confirmed asthma diagnosis. At the age of ten, 86 % of these cases (n = 158) and controls (n = 171) completed an ISAAC questionnaire concerning asthma history, symptoms and related conditions.

**Results:**

Different types of asthma symptoms were highly and significantly over-represented in the cases. Reported asthma heredity was significantly higher among the cases. No significant difference in reported allergic rhinitis or eczema as a child was found between cases and controls. No significant difference concerning social factors or environmental exposure was found between case and controls. Among the control group 4.7 % of the parents reported that their child actually had asthma. These are likely to be new asthma cases between the age of seven and ten and give an estimated asthma prevalence rate at the age of ten of 15.1 % in the studied cohort.

**Conclusion:**

A combination of medical verified asthma diagnosis through medical records and the use of self-reported symptom through the ISAAC questionnaire seem to be valid and reliable measures to follow-up childhood asthma in the local community. The asthma prevalence at the age of ten in the studied birth cohort is considerably higher than previous reports for Sweden. Both the high prevalence figure and allowing the three-year lag phase for further settling of events in the community point at the complementary roles of both hospital and primary care in the comprehensive coverage and control of childhood asthma in the community.

## Background

In the last decades, childhood asthma has risen dramatically and now forms a major and still increasing health problem, notably but not exclusively in the affluent parts over the world [1-4]. Large research efforts have therefore been directed to it, and especially the multinational International Study of Asthma and Allergies in Childhood (ISAAC) [4] has only in the last few years provided a wealth of additional data, e.g., on prevalence [5-12] and symptoms [8,13,14], which further document the worsening of the situation [15] in spite of increasing community awareness and number of children receiving inhaled steroids[11] and other treatment.

In an international study of 12-year old children in 1994, questionnaire-reported asthma-prevalence was found to range from 16.8 % in New Zealand, 12 % in Wales and 11.5 % in South Africa to only 4% in Sweden [16]. Support of these data and that questionnaires may not grossly over-estimate prevalence is found by another questionnaire study from Sweden in 1989 where a frequency of 5.1 % in 9000 rural children of age between 4–14 years was reported [17], whereas in 1988 the percentage of 7–8-year-old children in Northern Sweden with asthma diagnosed by a physician was found to be 6% [18]. A previous Swedish cumulative incidence investigation was reported for a defined region in 1992, where up to the age of 12–14 years, 5.3 % of an n = 1654 birth-year cohort were seen at the University Hospital with a clinically confirmed diagnosis of asthma [19].

Further examination of the epidemiology and natural history [20] of childhood asthma is therefore warranted, especially in infancy since it starts before the age of six in about 80–90 per cent of the cases. Hereby, comprehensive coverage in a well-defined area might complement the mainly large-scale surveys that are so far available.

Also the etiological aspects, i.e. the associations and determinants of the disorder are of high interest. Since before, factors like changes in housing conditions, broad-spectrum antibiotic use, viral infections, dietary habits, less out-door activities, young maternal age, season of birth, living in urban or rural areas, and social, cultural and economic conditions [21-28] have been discussed, but not considered sufficiently clarified [29]. The new, mainly ISAAC findings here provide a host of extra, though as a rule cross-sectional and scattered information, e.g., on food allergy [30], rhinitis [31-33], wheezing [33-37], reduced pulmonary function [34,35], atopy [31,34,36,38,39], virus infections [40], smoking [38,39,41], socioeconomic status [42], diet [31], behaviour problems [43], exposure to pets [38,44,45], indoor chlorinated swimming pools [46], per capita gross national product [47], and Caesarean section [48]. However, a more overall inventory of risk factors [49,50], especially according to the standards of a regular case-control study [51,52] is still rare.

The present account of the findings in a defined and representative geographic area, involving childhood asthma and related conditions, hospital and primary care services, and diagnosis and questionnaire assessments, should therefore be of value, especially since it emanates from a country with previously low childhood asthma prevalence in an international comparison.

The aims were to follow up at the age of ten the patterns of asthma symptoms and associations among children with a clinically diagnosed asthma in a sizeable urban-rural community and to compare them with demographic controls using a standardised questionnaire.

## Methods

In the representative, mixed urban-rural geographical region of Linköping in the county of Östergötland in Southern Sweden, with a population of about 150 000, all children (n= 2 104) born in 1990 at the University Hospital (which is the only somatic hospital in Linköping and where all births occurred) except those suffering neonatal death and those living outside the region, were recorded. For all of them still living in the region at the age of seven, the computerised medical records of the Department of Paediatrics at the University Hospital and at all 14 Primary Health Care (PHC) Units and at the two existing private Paediatrician Offices in the region were examined for the occurrence of the principal diagnosis asthma (ICD-9: 493). Secondary asthma-suspected or asthma-like symptoms were disqualified. Data of perinatal and obstetric factors as well as some social factors at baseline (1990) were obtained by investigations of the mothers' medical records at the University Hospital including check-ups in the PHC organisation of statements made by the mid-wife in the medical records during the pregnancy.

**Figure 1:**
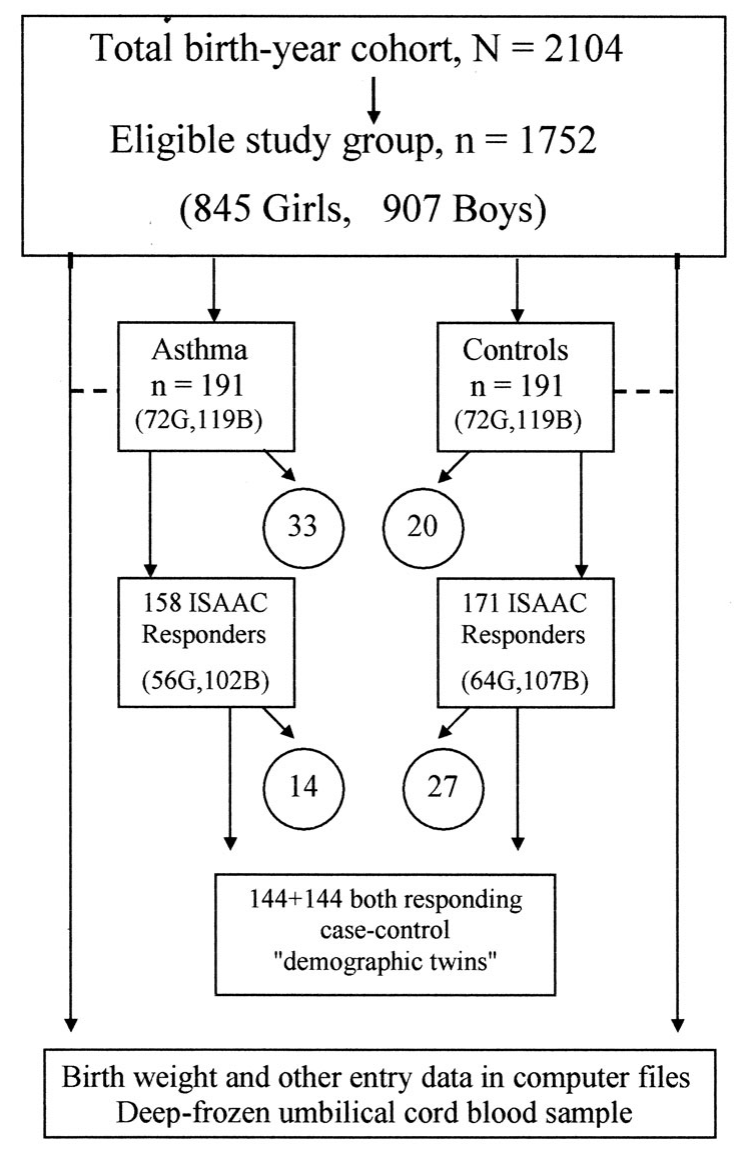
Participants in the study.

Children born 1990 in the region and still living in the region at the age of seven, was the inclusion criteria. At the age of seven n = 1752 children (n = 845 girls and n = 907 boys) met these criteria's as shown in figure 1. Practically all of the missing children at this follow-up had moved out of the region, only a few had deceased. Children born in 1990 that had moved in to the region after 1990 were excluded from the present study. In the follow-up at the age of seven, n = 191 children of this birth cohort were found in the medical records with a documented asthma diagnosis. In all, n = 41 cases of asthma (8.5 %) were identified in the urban girls and n = 31 (8.5 %) in the rural, whereas the corresponding figures for the urban and rural boys were n = 73 (14.2 %, relative risk (RR) to all girls 1.67, p < 0.005) and n = 46 (11.7%, RR to all girls 1.37, p = 0.051), respectively [53]. Together, these 191 cases gives a total cumulative asthma incidence at the age of seven of 10.9 %, overall significantly (p < 0.05) higher among the boys (13.1 %, n = 119) than the girls (8.5%, n = 72). Increased relative risks were also noted in children born in the autumn and winter, and in children born of the 392 mothers in the youngest maternal age bracket, <25 years [53,54].

To each of the identified asthma cases at the age of seven, a matched control (a child of the same sex with the nearest birth date i.e. "a demographic twin") was identified forming an equally large control group (n = 191). When the children with a documented asthma diagnosis were ten years old, their parents and their matched controls (total n = 382) were sent the International Study of Asthma and Allergies in Childhood (ISAAC) questionnaire [11] concerning asthma history, symptoms, related conditions, heredity, socio-economic factors and environmental exposure. The response rate to this postal questionnaire was 82.7 % (n = 158) in the asthma cases and 89.5 % (n = 171) in the controls, in total a response rate of 86.6 %. The number of matched twins gained with both a case and a control respondent was limited to n = 144 cases and n = 144 controls. However, the findings of the ISAAC questionnaire in this report are presented for all the responders from the case group (n = 158) and the control group (n = 171), respectively, in total n = 329 responders.

All data were stored and computerised in a common database and statistically analysed using the SPSS-program. In the statistical analysis, differences were assessed by the chi^2^-method. The estimation of cumulative asthma incidence at the age of ten in the cohort was based on an assumption that the same proportion of new cases occurred in the whole cohort between the age of seven and ten as among the controls.

## Results

There were no significant differences concerning social factors like; having younger or older siblings, social class and living conditions between the cases and controls, as presented in Table 1. The proportion of smoking mothers was higher (p = 0.007) among the cases (33.6 % smoking mothers) than among the controls where 20.5 % of the mothers were smokers.

**Table 1:** Comparison of background factors between cases and controls.

	Case (n = 158)		Control (n = 171)		
	n	%	n	%	p-value
**Gender**					
Male	102	64.5	107	62.5	
Female	56	35.4	64	37.4	0.71
**Having older siblings**	99	62.6	108	63.1	0.37
**Having younger sibling**s	92	58.2	93	54.3	0.45
**Social class of father**					
1	21	13.2	20	11.7	0.85
2	51	32.2	57	33.3	
3	75	47.5	87	50.8	
**Social class of mother**					
1	8	5.0	7	4.0	0.35
2	69	43.6	62	36.2	
3	81	51.2	100	58.4	
**Living conditions during the child's first 3 years**					
Countryside	61	38.6	79	46.2	0.16
City	92	58.2	92	53.8	
Mixed	5	3.2	0	0.0	
					
Villa/house	102	64.5	116	67.8	0.46
Apartment	53	33.5	49	28.6	
Mixed	3	1.8	6	3.5	
**Living conditions at the age of 10**					
Countryside	71	44.9	89	52.0	0.16
City	83	52.5	82	47.9	
Mixed	4	2.5	0	0.0	
					
Villa /house	118	74.7	135	78.9	0.29
Apartment	36	22.7	35	20.4	
Mixed	4	2.5	1	0.5	
**Smoking habits of father**					
Non-smoker	117	74.1	140	81.8	0.22
Smokes 0–9 cig/day	13	8.2	15	8.8	
Smokes 10–20 cig/day	19	12.0	11	6.4	
Smokes >20 Cig /day 2	1.3	5	2.9		
**Smoking habits of mother**					
Non-smoker	105	66.4	136	79.5	0.007
Smokes 0–9 cig/day	24	15.2	13	7.6	
Smokes 10–20 cig/day	27	17.1	20	11.7	
Smokes >20 Cig /day	2	1.3	2	1.2	
**Smoking habits of other members of the household**					
Non-smoker	152	96.2	164	95.9	0.34
Smokes 0–9 cig/day:	4	2.5	6	3.5	
Smokes 10–20 cig/day:	0	0.0	1	0.5	
Smokes >20 Cig /day	2	1.2	0	0.0	
**Smoking at home during the first 3 years**					
No:	128	81.0	143	83.6	0.53
Yes:	30	18.9	28	16.3	

There were no differences between cases and controls in self-reported in-door exposure of mist, mould or dry air inside the house. Exposure to pet animals like cat, dog, and animals with furs or cage birds was almost the same between cases and controls (not shown).

Among the cases, 39.3 % reported asthma heredity in the family and this was significantly lower 26.4% (p = 0.007) among the controls, as presented in Table 2. Heredity for allergic rhinitis in the family was reported by 57.6 % among the cases and 46.8 % among the controls. Almost 50 % in both cases and controls reported heredity for eczema as a child in the family.

**Table 2:** Comparison of heredity between cases and controls.

	Case (n = 158)		Control (n = 171)		
	n	%	n	%	p-value
**Asthma**					
No	96	60.7	126	73.6	0.007
Father	12	7.6	7	4.1	
Mother	12	7.6	7	4.1	
More than one in the family	18	11.4	5	2.9	
Sibling	20	12.6	26	15.2	
					
**Allergic rhinitis**					
No	67	42.4	91	53.2	0.10
Father	16	10.1	20	11.7	
Mother	29	18.4	17	9.9	
More than one in the family	34	21.5	28	16.3	
Sibling	12	7.6	15	8.8	
					
**Eczema as a Child**					
No	80	50.6	87	50.8	0.97
Father	8	5.0	6	2.9	
Mother	15	9.5	17	9.9	
More than one in the family	22	13.9	25	14.6	
Sibling	33	20.8	36	21.0	

Table 3 gives an overview of past and present symptoms and signs of asthma. It is seen that they are as such highly significantly over-represented in the cases. Wheezing or whistling in the chest at any time in the past was reported by around 75 % of the asthma cases. Even among the controls almost one fourth reported this symptom, while its frequency during last year was 26.6 versus 5.8 %. In around one fourth of the cases it was reported that the chest of the child had sounded wheezy after or during exercise during the last year, in comparison with 5% of the controls. Sleep and speech disturbances were exceptional and more frequent among the cases, but not significantly so. However, dry cough at night during the last 12 months, not associated with a cold or chest infection, was significantly (p < 0.0001) more common among the cases (33%) in comparison with the controls (11%). Only in a few children, but significantly more among the cases (p = 0.007), it was reported that they had been blocked in the chest or experienced mucous cough ≥ 4 days per week during a period of at least 3 months per year.

**Table 3:** Comparison of symptoms between cases and controls.

	Case (n = 158)		Control (n = 171)		
	n	%	n	%	p-value
**A. Wheezing or whistling in the chest at any time in the past**					
Yes	116	73.4	40	23.4	<0.0001
No	42	26.6	131	76.6	
					
**B. Wheezing or whistling in the chest in the last 12 months**					
Yes	42	26.6	10	5.8	<0.0001
No	116	73.4	161	94.1	
					
**C. How many attacks of wheezing in the last 12 months**					
None	113	71.5	161	94.1	<0.0001
1–3	25	15.8	5	2.9	
4–12	18	11.3	5	2.9	
More than 12	2	1.3	0	0.0	
					
**D. Disturbed sleep due to wheezing in the last 12 months**					
Never woken with wheezing	137	86.7	163	95.3	0.08
Less than one night per week	20	12.6	6	3.5	
One or more nights per week	1	0.6	2	1.1	
					
**E. Limiting speech to only one or two words at a time between breaths last 12 months**					
Yes	5	3.1	3	1.7	0.40
No	153	96.8	168	98.2	
					
**F. Has the child's chest sounded wheezy after or during exercise last 12 months**					
Yes	42	26.6	9	5.2	<0.0001
No	116	73.4	162	94.7	
**G. Dry cough at night (not associated with a cold or chest infection) last 12 months**					
Yes	52	32.9	19	11.1	<0.0001
No	106	67.1	152	88.9	
					
**H. Blocked in chest or mucous cough ≥ 4 days/week during in total ≥ 3 months per year**					
Yes	11	6.9	2	1.1	0.007
No	147	93.0	169	98.9	
					
**I. Problem with sneezing or a runny or blocked nose without a cold or the flu**					
Yes	70	44.3	37	21.6	< 0.0001
No	88	55.7	134	78.4	
					
**J. Itchy rash coming and going for at least 6 months**					
Yes	49	31.0	35	20.5	0.03
No	109	68.9	136	79.5	

Problem last year with sneezing or a runny or blocked nose without a cold or flu was reported by 44 % of the asthma cases and by just over 20 % of the controls. Coming and going itchy rash during last 6 months was significantly (p = 0.03) more frequently reported in the cases (31%) in comparison with the controls (20%).

In the follow-up ISAAC-questionnaire the parents were asked if their child ever had asthma, eczema or hay fever. The results are presented in Table 4. Among the asthma cases 46.8 % also reported eczema, while the corresponding figure for the controls was 39.2 %. Hay fever was reported for 21.5 % of the asthma cases and 14 % for the controls. In the cases with a well-documented asthma diagnosis, 39.2 % of the parents answered that their child never had asthma. On the other hand, among the control group 4.7 % (8 children; 3 girls and 5 boys) of the parents reported that their child actually had asthma. These 8 children are possibly new asthma cases that had occurred between the age of seven and ten. If so, one can estimate an incidence rate at the age of ten for the studied cohort. The rate at the age of seven in the cohort was 10.9 %. The eight potentially new cases represent 4.7 % of the control group (n = 171) and if the same proportion (4.7 %) is applied for the whole cohort (4.7 % of n = 1 561 children) it gives an estimated additional number of n = 73 new asthma cases between the age of seven and ten, which gives an estimated cumulative incidence rate at the age of ten of 15.1 %.

**Table 4:** Comparison of previous diseases between cases and controls.

	Case (n = 158)		Control (n = 171)		
	n	%	n	%	p-value
**Has your child ever had asthma?**					
Yes:	96	60.7	8	4.7	<0.0001
No:	62	39.2	163	95.3	
					
**Has your child ever had eczema?**					
Yes:	74	46.8	67	39.2	0.16
No:	84	53.2	104	60.8	
					
**Has your child ever had hay fever?**					
Yes:	34	21.5	24	14.0	0.075
No:	124	78.5	147	85.9	

A comparison of symptoms at the age of ten between the eight potentially new asthma cases and the previously confirmed asthma cases and controls is shown in Table 5. It is seen that in terms of symptoms and associations they appear as genuine cases and hence also diagnostically qualify as potentially new cases between the age of seven and ten from the part of the initial non-asthma cohort.

**Table 5:** Symptoms (A. to J. as in Table 3) among potentially new asthmacases in comparison with previously identified asthma cases and controls.

		New asthma cases" (n = 8)		Asthma cases (n = 158)		Controls (n = 163)		
		n	%	n	%	n	%	p-value
**A.**	Yes	8	100.0	117	73.6	33	20.1	<0.0001
**B.**	Yes	4	50.0	42	26.4	7	4.3	<0.0001
**C.**	Yes, 1 or more	4	50.0	45	28.3	7	4.2	<0.0001
**D.**	Yes	3	37.5	21	13.2	5	3.0	<0.0001
**E.**	Yes	1	12.5	5	3.1	2	1.2	0.09
**F.**	Yes	4	50.0	42	26.4	6	3.7	<0.0001
**G.**	Yes	2	25.0	53	33.3	17	10.4	<0.0001
**H.**	Yes	0	0.0	11	6.9	2	1.2	0.03
**I.**	Yes	4	50.0	70	44.0	34	20.7	<0.0001
**J.**	Yes	1	12.5	49	30.8	34	20.7	0.08

## Discussion

When the diagnosis of childhood asthma in the large majority of recent studies is based upon the ISAAC questionnaire, which has been tested with a sensitivity in relation to various standards between 64–76 [5] and 75–87 [6] but generally low specificity and predictive values, the obtained prevalence is remarkably high; from about 6.7 – 10.2 in Brazilian schoolchildren [8], 9 % in Texas [5], 7.2 – 9.6% in Palestine [7], 16.3 % in Australian indigenous children [6], and 22.3 % in the northeast of England [9]. This is clearly higher than corresponding data from, e.g., 1994 [16], and calls for further study including clinical and questionnaire comparison, where we think that our project is of value both in terms of scale, relevance and representativity. It covers a whole birth-year cohort in a sizeable and epidemiologically well defined, mixed urban-rural region. In the initial study group, it is based on the totality of principal asthma diagnosis clinically confirmed and documented in the health services network of Linköping, i.e., not only at the University hospital but also in the private paediatrician and public PHC organisation. In the follow-up it is complemented by the thoroughly validated ISAAC questionnaire, allowing assessment and cross-comparison of symptoms and some relevant proxy associations in both cases and controls, as well as transfer of by these means identified new cases from the latter to the former group leading to a complete 0–10-year cumulative incidence estimation in the order of 15.1 %.

We believe that strength of our study is its case-control design in which the ISAAC questionnaire was complementary and could be followed up clinically. We therefore believe that the 0–7-year incidence for childhood asthma of around 11 % and 0–10-year around 15 % reflects the contemporary situation in the studied Swedish region. Since only principal asthma diagnosis was included, there are few false positives; in fact, because the clinical diagnosis of asthma is regularly used as standard for sensitivity and specificity calculations, these expressions are not appropriate here. However, even if it is as such methodologically acceptable to estimate asthma incidence at age 10 by adding to the rate at age seven the new cases that developed in the control group, the further approximation in this based upon the data in the questionnaire cases presents a certain limitation to the findings. A more detailed clinical and therapeutic assessment of the material including also the cases reported to be free of asthma at the follow-up should be warranted in coming studies.

This apparent remission in a proportion of the cases is interesting per se and somewhat at variance with the stereotype of asthma as a permanent chronic illness with, in analogy with other long-term childhood diseases, implicitly more serious prognosis the earlier the onset. The children in the ten-year follow-up reported to be free of asthma, in consequence represent an interesting category. As such, parental reports of childhood asthma have been found to be reliable [55,56], so this question clearly warrants separate examination because elucidation of the patterns in that group might give additional preventive and therapeutic clues.

However, also the patterns in the persisting cases provide several but still often quite puzzling hints to that end. One must again ask what the reasons are: of the disorder itself as well as of its steep world-wide rise of late. It is apparent, that expanded health services have not served to alleviate the problem. The studied Swedish region has a well developed infant and school health organization – yet childhood asthma escalates almost uncontrollably as judged from the obtained incidence data; from 5.3 % in 1992 [19] to the current estimated 15.1 %. In addition there are high and rising figures for symptoms like rhinitis and wheezing which, as well known in previous studies [31,37], might be an associated but also an extra problem as inferred from the overlap between cases and controls in table 3.

The higher frequency in boys is well known [5-12,53], too, and again one might ask: Why? Implicitly it somewhat goes against the tacit assumption that overly cleanliness predisposes to asthma. On the other hand, the seasonal variation with autumnal, in the Northern Hemisphere September, October and November incidence peaks is likewise established [53], and in that connection, boys might be more exposed to outdoor, plausibly physiological strains, notably cold, which from earlier studies showing higher childhood asthma prevalence in Northern than in Southern parts of Sweden [57] would seem to be of importance.

Urban dwelling were other factors that showed weakly significant differences between cases and controls in this study, whereas we could not verify associations with socio-economic class or pet animal or indoor allergen exposure. Smoking exposure in the family, especially from the mother, is confirmed to be of importance in study as in previous follow-up [54]. A relation to asthma and to a lesser extent hay fever heredity was confirmed, however, and stemming from such a complete and sizeable material indicates that one important line of future research, which we are following also in our project, goes into a more specific constitutional and genetic direction [52,54].

As expected, established symptoms of asthma were significantly over-represented in the clinically diagnosed cases, but there was a strong overlap to the controls in this study. This supports that questionnaires alone may serve well as screening instruments but constitute somewhat of a circle argument in establishing an asthma diagnosis. It is true that one can discern highly specific constellations of symptoms and signs, but they are rare so do not contribute much to the operative strength. It has been pointed out in the literature, that such shortcomings strongly call for more stringent case-control studies of the type here reported [51,52], and from which further investigations on the individual level are then of equally large interest. Reciprocally, similar applies to the non-correlation to common cold, and, more intriguingly, eczema that we noted in this study.

We think that the further study potential is strengthened by the completeness of the material. Instrumental for this was the engagement also of the non-hospital services in its recruitment. Even when the University Hospital provided the bulk of cases, the virtually total population coverage of the PHC organisation provided the otherwise missing. Moreover, the high response frequency to the ISAAC questionnaire that we used in a confirming sense, both shows the high parental concern understandably given to asthma in their child and ensures a maximal degree of completeness of the data.

## Conclusion

A combination of medical verified asthma diagnosis through medical records and the use of self-reported symptom through the ISAAC questionnaire seem to be valid and reliable measures to follow-up childhood asthma in the local community. The asthma prevalence at the age of ten in the studied birth cohort is considerably higher than previous reports for Sweden. Both the high prevalence figure and allowing the three-year lag phase for further settling of events in the community point at the complementary roles of both hospital and primary care in the comprehensive coverage and control of childhood asthma in the community.

As inferred from the symptom spectrum presented in this report, especially the therapeutic aspects call for increased study and the findings support that equal alert is warranted during the whole childhood period. The follow-up indicated an almost five percent additional incidence in the period 8–10 years of age. It is thus tempting to conclude that there is a continuous interplay between external and constitutional factors, which are so far only quite scarcely known and accordingly warrant extensive further research. Also applying to the considerable fraction of cases, as judged from the parental report in this study, that are cured from the disease, such studies are under way.

## Competing interests

The author(s) declare that they have no competing interests.

## Authors' contributions

ER, ÅF and TF conceived and designed the study, participated in the collection, statistical analysis and interpretation of data and drafted the manuscript. ET and OZ participated in the analysis and interpretation of data and draft of the manuscript. All authors read and approved the final manuscript.

## Pre-publication history

The pre-publication history for this paper can be accessed here:


